# The efficacy and tolerability of artemisinin-piperaquine (Artequick®) versus artesunate-amodiaquine (Coarsucam™) for the treatment of uncomplicated *Plasmodium falciparum* malaria in south-central Vietnam

**DOI:** 10.1186/1475-2875-11-217

**Published:** 2012-06-28

**Authors:** Nguyen Xuan Thanh, Trieu Nguyen Trung, Nguyen Chinh Phong, Huynh Hong Quang, Bui Dai, G Dennis Shanks, Marina Chavchich, Michael D Edstein

**Affiliations:** 1 Military Institute of Hygiene and Epidemiology, Hanoi, Vietnam; 2 Institute of Malariology, Parasitology and Entomology, Qui Nhon, Vietnam; 3 Australian Army Malaria Institute, Enoggera, Brisbane, QLD, 4051, Australia

**Keywords:** Malaria, *Plasmodium falciparum*, Artemisinin, Piperaquine, Artesunate, Amodiaquine

## Abstract

**Background:**

In Vietnam, the artemisinin-based combination therapy (ACT) of dihydroartemisinin-piperaquine is currently used for first-line treatment of uncomplicated *Plasmodium falciparum* malaria. However, limited efficacy and tolerability data are available on alternative forms of ACT in Vietnam in case there is a reduction in the susceptibility of dihydroartemisinin-piperaquine. A study was conducted to compare the efficacy and tolerability of two fixed-dose formulations of ACT, artemisinin–piperaquine (Artequick®, ARPQ) and artesunate-amodiaquine (Coarsucam™, ASAQ) for the treatment of *P. falciparum* malaria in south-central Vietnam.

**Methods:**

A randomized, open-label trial was conducted comparing the efficacy of a two-day regimen of ARPQ (~2.8 mg/kg artemisinin plus ~17.1 mg/kg of piperaquine per day) and a three-day regimen of ASAQ (~4.7 mg/kg of artesunate plus ~12.6 mg/kg of amodiaquine per day) for the treatment of children and adults with uncomplicated falciparum malaria. Primary efficacy endpoint was day 42, PCR-corrected, parasitological cure rate. Secondary endpoints were parasite and fever clearance times and tolerability.

**Results:**

Of 128 patients enrolled, 63 were administered ARPQ and 65 ASAQ. Of the patients who completed the 42 days follow-up period or had a recurrence of malaria, 55 were on ARPQ (30 children, 25 adults) and 59 were on ASAQ (31 children, 28 adults). Recrudescent parasitaemia was PCR-confirmed for one patient in each treatment group, with cure rates at day 42 of 98% (95% CI: 88–100) for both forms of ACT. The median parasite clearance time was significantly slower in the ARPQ group compared with the ASAQ group (48 h *vs.* 36 h, *P*<0.001) and fever clearance times were shorter in the ASAQ group (12 h *vs.* 24 h, *P* = 0.07). The two forms of ACT were well tolerated with no serious adverse events.

**Conclusion:**

Both forms of ACT were highly efficacious in the treatment of uncomplicated *P. falciparum* malaria. Although the two-day course of ARPQ was equally as effective as the three-day course of ASAQ, parasite and fever clearance times were shorter with ASAQ. Further studies are warranted in different regions of Vietnam to determine the nationwide efficacy of ASAQ.

**Trial registration:**

Australian New Zealand Clinical Trials Registry Number, ACTRN12609000816257

## Background

In 2010, the World Health Organization (WHO) estimated that half of the world’s population was at risk of malaria, with an estimated 216 million cases and about 655,000 deaths [1]. For first-line treatment of uncomplicated *Plasmodium falciparum* malaria worldwide, WHO recommends artemisinin-based combination therapy (ACT), such as artemether-lumefantrine, artesunate-amodiaquine, artesunate-mefloquine, artesunate-sulphadoxine-pyrimethamine and dihydroartemisinin-piperaquine [2]. Most of these formulations of ACT are available as fixed-dosed co-formulations, facilitating improved adherence, convenience and prevention against misuse [3]. Of these, artemether-lumefantrine (Coartem®, three-day six-dose regimen, total dose ~12/72 mg/kg, respectively) and dihydroartemisinin-piperaquine (Artekin®, Duo-Cotecxin®, Eurartesim™, three-day three-dose regimen, total dose ~ 6/48 mg/kg, respectively) have been shown to be highly efficacious (day 42 cure rates >95%), safe and well tolerated in most studies conducted so far in Southeast Asia [4-8].

Since the mid-1990s, artesunate plus mefloquine (two and three-day regimens, total dose 12/25 mg/kg, respectively), usually given as separate tablets, has been the treatment of choice for multidrug-resistant falciparum malaria in Southeast Asia, particularly along the north-western border of Thailand, where mefloquine resistance is prevalent [9]. Recent studies, however, along the Thailand-Myanmar [10] and Cambodia-Thailand [11] border regions have shown evidence of modest resistance to artesunate plus mefloquine (three-day regimen), with a prolongation in parasite clearance times. Reduced PCR-adjusted 42-day cure rates of about 80% have also been reported in western and southern Cambodia following treatment with artesunate plus mefloquine [12,13]. These findings of emerging ACT-resistant *P. falciparum* malaria are of immense concern, as the Cambodia-Thailand border region has been the epicentre of anti-malarial drug resistance [14], which historically has spread westward from South Asia to Africa [5].

In Vietnam, the three-day fixed-dose combination regimen of dihydroartemisinin-piperaquine (Arterakine®) is now the recommended first-line ACT for the treatment of uncomplicated falciparum malaria [15]. Although dihydroartemisinin-piperaquine has proved to be well tolerated and highly efficacious against *P. falciparum* in Vietnam [16,17], there is limited published data on the efficacy and tolerability of other formulations of ACT in Vietnam. Because of the concern of reduced efficacy of artesunate plus mefloquine in neighbouring Cambodia, it is considered prudent to continue to evaluate alternative forms of ACT in Vietnam in case there is a decline in the efficacy of dihydroartemisinin-piperaquine. Recently, a three-day regimen of artesunate plus amodiaquine administered as separate tablets was highly efficacious (PCR adjusted 42-day cure rate of 98%) and well tolerated in the treatment of uncomplicated falciparum malaria in Vietnamese patients [17]. These encouraging findings with artesunate plus amodiaquine prompted an assessment of the efficacy and tolerability of a fixed-dose combination of artesunate-amodiaquine (Coarsucam™) given over three days at the same study site in Ninh Thuan Province, south-central Vietnam where the previous artesunate plus amodiaquine study was conducted. As a comparative treatment arm, a two-day regimen of artemisinin–piperaquine (Artequick®) was also evaluated as the manufacturer (Artepharm, Guangzhuo, People’s Republic of China) advocates high efficacy, low cost, low toxicity, and ease of administration with a short course of four tablets [18].

## Methods

### Study site, patients, and ethics

The study was conducted at Phuoc Chien Commune, Thuan Bac District, Ninh Thuan Province in south-central Vietnam, located about 300 km north of Ho Chi Minh City. The commune has a population of about 4,000 people, with most belonging to the Ra-glai ethnic group. Malaria transmission is low and occurs perennially with two peaks (May-June and October-November) [19]. *Anopheles dirus* and *Anopheles maculatus* are the main malaria vectors on the forested hill areas near the commune [20]. Study participants were recruited among patients presenting at the Phuoc Chien health station. Patients with suspected clinical malaria were screened and enrolled in the study if they met the following inclusion criteria: age 5–60 years; a blood slide confirmed *P. falciparum* mono-infection with a parasite density of 200–200,000 asexual parasites/μL; tympanic temperature ≥38°C at the time of enrolment or history of fever during the preceding 24 h and willingness to be followed-up for 42 days after starting treatment. Exclusion criteria were as follows: patients with symptoms and/or signs of severe malaria; evidence of another serious medical disease; a history of drug or alcohol abuse; anti-malarial treatment within the preceding seven days; mixed plasmodial infection or pregnant and lactating females. Written informed consent was obtained from each adult patient or from parents or legal guardians of enrolled children. The study was ethically approved by the Vietnam Ministry of Health, Vietnam People’s Army Department of Military Medicine and the Australian Defence Human Research Ethics Committee (Approval No. 507/08).

### Study design and treatment

In the open-label, randomized study the patients were sequentially allocated to the two treatment groups: artemisinin-piperaquine (ARPQ, each tablet contained 62.5 mg artemisinin and 375 mg piperaquine, Artequick®, Artepharm Co., Ltd, Guangzhou, China) and artesunate-amodiaquine (ASAQ, each tablet contained 100 mg artesunate and 270 mg amodiaquine, Coarsucam™, Sanofi-Aventis, Morocco), with the first patient receiving ARPQ, the second patient ASAQ, the third patient ARPQ, and so on. A weight-based regimen of ARPQ (2.8 mg/kg artemisinin plus ~16.7 mg/kg piperaquine per day for two days) and ASAQ (~4.4 mg/kg artesunate plus ~12.0 mg/kg amodiaquine per day for three days) based on a 45 kg adult was rounded up or down to the nearest half tablet. Both forms of ACT were administered under direct observation and the drugs were given with condensed milk. If a patient vomited the drugs within 30 min, a full dose was readministered. The rescue medication was a seven-day regimen of artesunate (4 mg/kg on the first day and then 2 mg/kg daily for six days) to be implemented if either parasitaemia had not declined by at least 75% within 48 h of commencing treatment with ARPQ or ASAQ or parasites were still present on day 7 after starting treatment. Patients with parasite reappearance were treated with the seven-day regimen of artesunate for *P. falciparum* and a three-day regimen of chloroquine (25 mg/kg) for *Plasmodium vivax* as per national policy.

Although artesunate plus amodiaquine given as separate tablets has been reported to be highly efficacious (cure rate 98%), the efficacy of the two-day regimen of ARPQ, with a 42 day follow-up period is unknown. WHO recommends that regardless of the treatment rate of failure, a minimum sample size of 50 patients in clinical trials is required in order for a study to be representative [21]. Thus, to achieve a minimum sample size of 50 patients for each treatment group, the aim was to recruit 130 patients, with up to 15% of patients calculated for loss during follow-up, withdraw, protocol violations or mixed infections at admission identified subsequently by PCR analysis [22]. The study was registered at Australian New Zealand Clinical Trials Registry [23], with a registration number of ACTRN12609000816257.

### Laboratory investigations and follow-up

Clinical assessment and parasite density counts were performed on days 0, 1, 2, 3, 7, 14, 21, 28, 35 and 42 or on any day of recurrent malaria infection. Thick and thin blood films were collected before and every 12 h after commencement of treatment until blood films were negative for three consecutive examinations. The Giemsa-stained blood films were examined by two microscopists and parasitaemia was quantified by examination of thick film fields (magnification × 1,000) against 200 leukocytes, assuming a total leukocytes count of 8,000/μL. Parasite clearance time was the time in hours from starting treatment until the asexual parasite count fell below detectable levels in thick blood films. Patient’s tympanic body temperature was measured immediately before and then every 12 h after starting treatment until their temperature was <38°C for two consecutive days. Fever clearance time was the time in hours from starting treatment until the patient’s tympanic body temperature remained <38°C for more than 48 h.

Blood spots on filter paper (3MM, Whatman) were collected at the same time as blood smears. PCR parasite genotyping was done to confirm plasmodial species using the QIAamp DNA mini kit (Qiagen, Cat. #51304) for DNA extraction, single round PCR detection and identification of the four species, *P. falciparumP. vivaxPlasmodium ovale* and *Plasmodium malariae* using specific PCR primers [24]. In order to differentiate reinfection from recrudescence, multiplex PCR analysis was performed on paired samples of *P. falciparum* DNA (day 0 and day of recurrence of parasitaemia) for allelic variation at three loci (merozoite surface proteins 1 and 2, and glutamate rich protein). If at least one allele of the recurrent parasitaemia was different to the before treatment parasitaemia, the infection was considered a reinfection.

Drug tolerability was assessed clinically. An adverse event was defined as any sign or symptom that occurred or became severe during the study regardless as to whether it was related to the medication. Adverse events were recorded by the physician at each drug administration and patients where asked to respond to the non-leading question ‘*How do you feel since you took the last tablets?*’ Since malaria infection, particularly during the acute phase can cause adverse effects such as nausea, abdominal pain, headache and dizziness, which often makes it difficult to distinguish disease effects from drug effects [25], no causal association was made between the adverse events and the ACT.

Adult patients were invited to provide a blood sample for drug analysis on day 7 after commencement of treatment. Blood (5 mL) was obtained by venipuncture, transferred to heparinized tubes and centrifuged at 1,400 *g* for 5 min. The separated plasma was transferred to Nunc cryopreservation tubes and stored at −25°C. The plasma samples were later transferred on dry-ice to the Military Institute of Hygiene and Epidemiology in Hanoi and then shipped to the Australian Army Malaria Institute in Brisbane, where they were stored at −80°C until analysis.

### Drug analysis

The reference compounds of piperaquine, amodiaquine, desethylamodiaquine and amodiaquine anologue were donated by WWARN [26]. Plasma piperaquine concentrations were measured using solid phase extraction with high-performance liquid chromatography (HPLC) [27,28]. The interassay precision (i.e. coefficients of variation) for piperaquine was 10.3% at 10 ng/mL, 6.8% at 100 ng/mL and 6.7% at 500 ng/mL. The lower limit of quantification (LLOQ) for piperaquine was 5 ng/mL using 0.5 mL of plasma.

Plasma amodiaquine and its principal biologically active metabolite, desethylamodiaquine concentrations were measured by HPLC using a Agilent 1100 Series consisting of Pump Model 1100-G1310A, Autosample Model 1100-G131A and Absorbance UV/VIS Detector Model G1314A set at 342 nm. The column used was a Zorbax SB-CN cartridge (250 x 4.6 mm, 5 μm USSF011231, Agilent) with Zorbax SB-CN (12.5 x 4.6 mm, 5 μm USSM003377) guard column. The mobile phase consisted of acetonitrile: 1 M sodium perchlorate: 0.1 M phosphate buffer pH 2 (16:1:83, v/v/v) with a flow rate of 1 mL/min. Samples were prepared for analysis as follows: to a 10 mL polypropylene tube were added plasma (500 μL), amodiaquine analogue (internal standard, 100 μL of 50 ng/mL) and 1 mL of acetonitrile. The contents were vortexed for 30 sec followed by the addition of 2 mL ammonia (specific gravity 0.88) and 5 mL methyl *t*-butyl ether. The contents were mixed for 20 min, centrifuged (1,600 *g* for 10 min) and the organic phase transferred to a new polypropylene tube. The organic phase was evaporated at 40°C using instrument grade air and the residue was reconstituted with 150 μL of mobile phase of which 50 μL was injected onto the HPLC. The retention times were about 6.5 min for desethylamodiaquine, 8.5 min for amodiaquine and 11.0 min for the internal standard. The interday precision was 9.6% at 5 ng/mL, 2.4% at 50 ng/mL and 0.4% at 500 ng/mL for amodiaquine. Corresponding interday precision for desethylamodiaquine were 4.0%, 6.2% and 6.0%. The extraction efficiencies were 76% and 71% for amodiaquine and desethylamodiaquine, respectively. The LLOQ for amodiaquine and desethylamodiaquine was 5 ng/mL.

### Statistical analysis

All data were analysed using SigmaStat (version 3.0 Jandel Scientific, CA, USA) and STATA 10.0 (StataCorp LP, College Station, Texas, USA). Descriptive statistics were used to summarize baseline values and demographic data. Normally distributed and nonnormally distributed data were compared using the Student’s *t*-test and the Mann–Whitney *U* test, respectively. The primary efficacy endpoint was PCR-adjusted 42-day cure rates after starting treatment. Secondary endpoints were parasite and fever clearance times and the occurrence of adverse events. Demographic and efficacy data were assessed by means of a per-protocol analysis, with recipients of rescue treatment counted as failures and new infections as cured. A Kaplan-Meier survival analysis with a log-rank test for statistical significance was applied. Differences in proportions were compared using the *χ*^2^ or Fisher’s exact test. Statistical significance was defined as a *P*<0.05.

## Results

**Figure 1:**
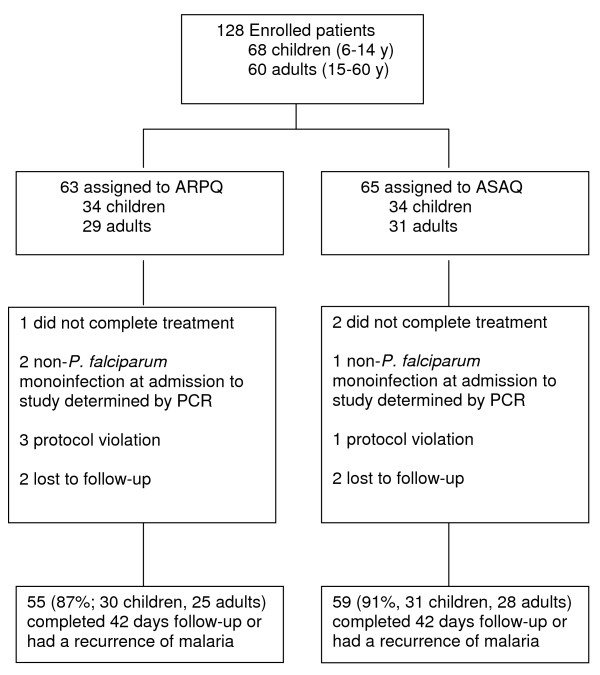
**Trial profile.** ARPQ: artemisinin-piperaquine; ASAQ: artesunate-amodiaquine.

The study was conducted between May 2008 and December 2009, with 128 patients enrolled consisting of 63 patients on ARPQ and 65 patients on ASAQ (Figure 1). Overall, eight patients in the ARPQ group and six patients in the ASAQ group did not fulfill the per-protocol requirements. At enrolment, the two treatment groups had similar demographic and clinical characteristics (Table 1). Of the patients recruited, 54% (34/63) on ARPQ and 52% (34/65) on ASAQ were children (<15 years of age). The geometrical mean parasitaemia was comparable between the two treatment groups (17,912 parasites/μL for ARPQ and 16,776 parasites/μL for ASAQ, *P* = 0.84). For both treatments, the geometrical mean parasitaemia was about two-fold higher in children compared with adults, but the differences were not significant (ARPQ - *P* =0.13; ASAQ - *P* = 0.09). At the time of admission, 64% (35/55) of patients on ARPQ and 59% (35/59) on ASAQ were febrile.

**Table 1:** Baseline characteristics of the patients according to treatment as per-protocol

	**ARPQ**	**ASAQ**
**No. of patients**		
All patients	55	59
Children	30	31
Adults	25	28
**No. of males (%)**		
All patients	38 (69%)	42 (71%)
Children	21 (70%)	24 (77%)
Adults	17 (68%)	18 (64%)
**Mean age (y) (±SD)**		
All patients	18.1 (12.9)	18.9 (12.7)
Children	9.1 (3.2)	10.2 (2.9)
Adults	29.0 (11.8)	28.5 (12.5)
**Mean weight (kg) (±SD)**		
All patients	31.1 (13.0)	34.0 (12.3)
Children	21.8 (7.6)	24.4 (7.6)
Adults	43.6 (6.7)	44.6 (6.2)
**Mean temperature (°C) (±SD)**		
All patients	38.6 (1.0)	38.3 (1.1)
Children	38.6 (0.9)	38.2 (1.2)
Adults	38.5 (1.3)	38.4 (1.0)
**No. (%) of patients with temp ≥38°C**		
All patients	35 (64%)	35 (59%)
Children	21 (70%)	18 (58%)
Adults	14 (56%)	17 (61%)
**Geometrical mean asexual*Plasmodium falciparum*/μL (range)**		
All patients	17,912 (617–162,527)	16,776 (590–114,550)
Children	23,785 (617–135,173)	23,522 (1,709-142,345)
Adults	12,746 (912–162,527)	11,450 (590–114,550)

Adults and children received comparable doses in mg/kg body weight of ARPQ or ASAQ. For the two-day regimen of ARPQ, adults were administered daily a median (interquartile range, IRQ) of 2.8 mg/kg (2.5 to 3.1) artemisinin and 17.0 mg/kg (15.0 to 18.8) piperaquine. Corresponding values for children were 2.9 mg/kg (2.2 to 3.2) artemisinin and 17.3 mg/kg (13.4 to 19.2) piperaquine. For the three-day regimen of ASAQ, adults were administered daily 4.4 mg/kg (4.1 to 4.8) artesunate and 11.8 mg/kg (11.0 to 13.0) amodiaquine. Corresponding values for children were 5.0 mg/kg (4.2 to 5.3) artesunate and 13.5 mg/kg (11.3 to 14.4) amodiaquine.

Treatment outcomes as per-protocol analysis for the two treatment groups are summarized in Table 2. The median parasite clearance time was significantly slower in the ARPQ group compared with the ASAQ group (48 h *vs.* 36 h, *P*<0.001) and parasite clearance tended to be faster in adults than in children (ARPQ, 36 h *vs.* 48 h, *P* = 0.02 and ASAQ, 24 h *vs.* 36 h, *P* = 0.03). The median fever clearance time was slower in the ARPQ group than in the ASAQ group but the difference was not significant (24 h *vs.* 12 h, *P* = 0.07).

**Table 2:** **Treatment outcomes as per**-**protocol after two-day course of artemisinin-piperaquine [ARPQ, children (n = 30), adults (n = 25)] and a three-day course of artesunate-amodiaquine [ASAQ, children (n = 31), adults (n = 28)] in Vietnamese patients with*Plasmodium falciparum*malaria**

		**ARPQ**	**ASAQ**	***P*value**
Parasite clearance time, median h (IQR)				
	All patients	48 (12–72)	36 (24–72)	<0.001
	Children	48 (24–72)	36 (24–72)	<0.001
	Adults	36 (12–60)	24 (24–48)	<0.001
Fever clearance time, median h (IQR)				
	All patients	24 (12–48)	12 (12–48)	0.07
	Children	24 (12–48)	12 (12–48)	0.06
	Adults	12 (12–24)	12 (12–48)	0.91
Recrudescence within 42 days, n/N (%)		1/55 (1.8%)	1/59 (1.7%)	0.51
Reinfection within 28 days, n/N (%)		0/55 (0%)	2/59 (3.4%)	0.51
Reinfection within 42 days, n/N (%)		7/55 (12.7%)^a, b^	9/59 (15.3%)^b^	0.91

^a^ Mixed infection (*Plasmodium falciparum* and *Plasmodium vivax*) was identified in 1 of 7 ARPQ recipients.

^b^*Plasmodium vivax* was identified in 3 of 7 recipients for both treatment groups.

IQR - Interquartile range.

For each treatment group only one patient had a recrudescence by day 42 of follow-up. The patient in the ARPQ group was a 14-year old male and at admission was febrile with a parasitaemia of 7,096 parasites/μL. His parasite clearance time was 48 h and his daily dose of artemisinin and piperaquine were 3.3 mg/kg and 19.7 mg/kg, respectively, which was slightly higher than the corresponding median doses of 2.8 mg/kg and 17.1 mg/kg for the cured children. He was diagnosed with a recrudescence on day 12 after starting treatment. In contrast to the ARPQ failure, the patient in the ASAQ group was a 28-year old male and at admission was febrile with a parasitaemia of 23,092 parasites/μL. His parasite clearance time was 36 h and his daily dose of artesunate and amodiaquine were 3.6 mg/kg and 9.8 mg/kg, respectively, which was less than the corresponding median doses of 4.4 mg/kg and 11.9 mg/kg for the cured adults. He was diagnosed with a recrudescence on day 33 after commencing treatment.

The PCR-adjusted cure rates were 98% for the ARPQ group (95% CI: 88%-100%) and 98% (95% CI: 88%-100%) for the ASAQ group (*P* = 0.96). Seven patients in the ARPQ group and nine patients in the ASAQ group had reinfections over the 42-day follow-up period. When combining recrudescences and reinfections, the cure rates were 87% (95% CI: 71%-91%) for ARPQ and 85% (95% CI: 73%-92%) for ASAQ (*P* = 0.70). During the first 28 days of follow-up, no patient on ARPQ had a reinfection, whereas two patients on ASAQ were diagnosed with new infections of *P. falciparum* on days 21 and 28. For each treatment group, three patients with reinfections were diagnosed with *P. vivax* malaria. Although not used in the analysis, of the protocol violations, one patient in each treatment group had a parasitaemia greater than 200,000 parasites/μL and both were successfully treated. Furthermore, the three patients with mixed infections of *P. falciparum* and *P. vixax* malaria at admission (two on ARPQ and one on ASAQ), based on subsequent PCR analysis, none had a recurrence of malaria during the follow-up period.

None of the patients had a serious adverse event and both treatments were well tolerated. The most common adverse events reported before and after treatment are shown in Table 3 and they were generally mild in intensity. The percentage of patients reporting adverse events was comparable between the two treatment groups before and after starting treatment. There was a marked decline in adverse events 24 h after the first dose of each ACT and by 48 h after commencing treatment most patients were free of adverse events.

**Table 3:** **Adverse events in patients who completed the two-day course of artemisinin-piperaquine (ARPQ, n = 62) and the three-day course of artesunate**-**amodiaquine (ASAQ, n = 63) in Vietnamese patients with*Plasmodium falciparum*malaria**

**ARPQ**	**Before treatment**	**24 h after 1**^**st**^**dose**	**24 h after 2**^**nd**^**dose**	**48 h after 2**^**nd**^**dose**
Fever	88.7% (55/62)	29.0% (18/62)	1.6% (1/62)	NR
Headache	87.1% (54/62)	48.4% (30/62)	1.6% (1/62)	1.6% (1/62)
Nausea	29.0% (18/62)	3.2% (2/62)	NR	NR
Stomach pain	1.6% (1/62)	NR	NR	NR
Vomiting	1.6% (1/62)	NR	NR	NR
Dizziness	27.4% (17/62)	3.2% (2/62)	NR	NR
Tiredness	61.3% (38/62)	25.8% (16/62)	3.2% (2/62)	1.6% (1/62)
Anorexia	43.5% (27/62)	9.7% (6/62)	1.6% (1/62)	1.6% (1/62)
**ASAQ**	**Before treatment**	**24 h after1**^**st**^**dose**	**24 h after 2**^**nd**^**dose**	**24 h after 3**^**rd**^**dose**
Fever	82.5% (52/63)	20.6% (13/63)	3.2% (2/63)	NR
Headache	95.2% (60/63)	46.0% (29/63)	3.2% (2/63)	NR
Nausea	41.3% (26/63)	19.0% (12/63)	1.6% (1/63)	NR
Stomach pain	NR	NR	NR	NR
Vomiting	3.2% (2/63)	3.2% (2/63)	NR	NR
Dizziness	25.4% (16/63)	3.2% (2/63)	NR	NR
Tiredness	69.8% (44/63)	30.2% (19/63)	3.2% (2/63)	NR
Anorexia	46.0% (29/63)	20.6% (13/63)	NR	NR

Seventy-two percent (18/25) of adults on ARPQ and 61% (17/28) of adults on ASAQ provided a blood sample at day 7 after starting treatment. The median day 7 plasma concentrations of piperaquine and desethylamodiaquine were 42 ng/mL (IQR, 23–63 ng/mL) and 54 ng/mL (IQR, 21–90 ng/mL), respectively. No amodiaquine was present at day 7.

## Discussion

The present study is the first to report on the efficacy and tolerability of ASAQ (Coarsucam™) in Vietnam. ASAQ was found to be highly efficacious (PCR-adjusted 42-day cure rate of 98%) in the treatment of uncomplicated *P. falciparum* malaria. The efficacy findings are in accord with an earlier study, at the same study site in south-central Vietnam, in which artesunate and amodiaquine were coadministered as separate tablets to patients [17]. When comparing the two studies, the admission geometric mean parasitaemia was similar (all patients: 14,074 parasites/μL *vs.* 16,776 parasites/μL). The median parasite clearance times, however, were significantly longer in patients given ASAQ compared with patients administered separate tablets (all patients 36 h *vs.* 24 h, *P* = 0.007) despite the ASAQ group receiving a slightly higher daily dose of the individual drugs (~4.7 mg/kg artesunate plus ~12.6 mg/kg amodiaquine *vs.* ~4.4. mg/kg artesunate plus ~10.6 mg/kg amodiaquine). Artesunate and amodiaquine given either as a fixed-dose combination or separate tablets was well tolerated in the Vietnamese patients and is in accord with the two drugs administered as different formulations (i.e. fixed *vs.* separate) for the treatment of *P. falciparum* in African children [29]. The comparable efficacy and tolerability findings of artesunate and amodiaquine given either as a fixed-dose combination or separately was not unexpected as the pharmacokinetics of the parent drugs and their principal biologically active metabolites have been shown to be similar in children with acute malaria [30].

Unlike the more commonly used fixed-dose combinations of ACT such as artemether-lumefantrine and dihydroartemisinin-piperaquine, there is limited data on the efficacy of ARPQ. The fixed-dose combination was developed by the Chinese as an alternative to dihydroartemisinin-piperaquine (Artekin® or Duo-Cotecxin®), with the manufacturer proposing the potential benefits of ARPQ’s cheaper cost of production and improve compliance (i.e. two-day *vs.* three-day regimen). In 2004, a comparative randomized efficacy study of two-day regimens of ARPQ (one dose daily per day) and dihydroartemisinin-piperaquine (Artekin® twice daily over two days) was carried out at Phuoc Chien Commune in 103 patients, with a 28-day cure rate of 100% for both forms of ACT [31]. However, the limitation of the study was that the follow-up period was far too short as a minimum of 42 days is required for forms of ACT [2,32] that have lengthy terminal elimination half-lives, such as piperaquine and mefloquine. Piperaquine has an elimination half-life of about 23–33 days [33-35] and for those patients who have failed dihydroartemisinin-piperaquine treatment, recrudescences tend to occur 28 days after starting treatment [6,36].

Similar to the study carried out in 2004, the shorter two-day regimen of ARPQ was highly efficacious, with a PCR-adjusted cure rate of 98%. This compares favourably with an earlier study at the same study site using three-day regimens of dihydroartemisinin-piperaquine (Arterakine®) and artesunate plus amodiaquine given as separate tablets [17]. As with other studies using piperaquine with dihydroartemisinin [6,17], ARPQ exhibited a better post-treatment prophylactic effect due to the longer elimination half-life of piperaquine (23–33 days) compared with the shorter half-life of desethylamodiaquine (9 days) [37] that is associated with ASAQ use. Although ARPQ’s extended post-treatment prophylactic effect is desirable the potential exists that low residual blood piperaquine concentrations may enhance the selection and development of piperaquine resistant parasites. Noteworthy, the three-day regimen of ASAQ cleared the *P. falciparum* infections in the recipients faster than in patients given the two-day regimen of ARPQ. In Thailand, Krudsood *et al.*[38] showed that in a dose-ranging study the two-day course of ARPQ was less effective than a three-day regimen of ARPQ (PCR-adjusted cure rates, 75% *vs.* 98%) in treating Thai patients with uncomplicated *P. falciparum* malaria, with a follow-up period of 28 days.

Presently, no marker exists that defines a consistent method for identifying reduced susceptibility of parasites to forms of ACT, particularly to the artemisinin component. Currently, prolongation of the parasite clearance time has been used as an important early warning sign of reduced artemisinin susceptibility. Most patients cleared their infections within 48 h of commencing their treatment with either ARPQ or ASAQ. Of the patients, 16.7% (9/54) on ARPQ and 3.4% (2/58) on ASAQ took up to 60 h to clear their infections. However, up to 72 h was required to clear parasites in one patient in each treatment group. Both patients were children of 12 years of age, with an admission parasitaemia of about 55,000 parasites/μL of blood. They were not administered a sub-optimal dose of the ACT, as they received a dose of each component above the median mg/kg body weight for the treatment group.

Although a two-day regimen may improve compliance such shorter dose regimens are not recommended by WHO [2]. The rapid acting artemisinins have a parasite reduction ratio (i.e. the fraction of infecting parasites cleared per asexual blood cycle) of >10^4^ and by themselves they need to be present at parasitological blood concentrations during at least three asexual blood stages to eliminate all parasites [39]. A limitation of the two-day regimen of ARPQ is that the artemisinin component is present for only one asexual cycle, as artemisinin has a short elimination half-life of about 2.3 h [40], which limits its exposure time to markedly reduce the parasitic load. Thus, ARPQ relies heavily on piperaquine to complete the clearance of all remaining parasites not inhibited by its partner drug. In regions where parasites are less susceptible to piperaquine, the two-day ARPQ regimen is likely to be less effective as the blood piperaquine concentration would be insufficient to eliminate all of the remaining parasites with the slower acting and less active piperaquine.

Plasma concentrations of piperaquine and desethylamodiaquine on day 7 have been found to correlate with drug exposure (i.e. area under the plasma drug concentration curve) and have been identified as major determinants of therapeutic response to dihydroartemisinin-piperaquine [41] and ASAQ [42]. The piperaquine day 7 concentration (median 42 ng/mL) in adults on the two-day course of ARPQ (total piperaquine base dose: 34 mg/kg) was not too dissimilar to Papuan adult patients (mean 50 ng/mL) treated with a three-day course of dihydroartemisinin-piperaquine (total piperaquine base dose: 30 mg/kg) [41]. The median plasma desethylamodiaquine concentration (54 ng/mL) in the Vietnamese patients is in accord with metabolite levels measured in African children (42 ng/mL - [42]) and adults (mean 62 ng/mL- [43]) at day 7 after initiation of treatment.

## Conclusions

ARPQ and ASAQ were highly and equally efficacious in the treatment of uncomplicated *P. falciparum* malaria in children and adults in south-central Vietnam. However, when comparing the two treatment regimens the three-day course of ASAQ resulted in a faster parasite and shorter fever clearance times than the two-day course of ARPQ. These findings warrant further investigation of the efficacy of ASAQ in other regions of Vietnam to ascertain the potential value of the ACT as an alternative option to dihydroartemisinin-piperaquine in case the first-line ACT starts to fail in Vietnam.

## Competing interests

The authors declare that they have no conflict of interest.

## Authors’ contributions

NXT, TNT, BD, GDS and MDE designed the study and developed the protocol. NCP and HHQ executed and coordinated the study. NCP, MC and MDE analysed and interpreted the data. MDE wrote the first draft of the paper. All authors read and approved the final manuscript.
